# Native Taylor/Non‐Taylor Dispersion–Mass Spectrometry (TNT‐MS) Allows Rapid Protein Desalting and Multiplexed, Label‐Free Ligand Screening

**DOI:** 10.1002/smtd.202500658

**Published:** 2025-05-07

**Authors:** Jonathan Eisert, Edvaldo Vasconcelos Soares Maciel, Verena Dederer, Aylin Berwanger, Henry J. Bailey, Ivan Đikić, Stefan Knapp, Martin Empting, Sebastian Mathea, Henrik Jensen, Frederik Lermyte

**Affiliations:** ^1^ Department of Chemistry Clemens‐Schöpf‐Institute of Chemistry and Biochemistry Technical University of Darmstadt Peter‐Grünberg‐Strasse 4 64287 Darmstadt Germany; ^2^ Institute of Pharmaceutical Chemistry Goethe University Max‐von‐Laue‐Str. 9 60438 Frankfurt am Main Germany; ^3^ Structural Genomics Consortium (SGC) Buchmann Institute for Life Sciences Max‐von‐Laue‐Str. 15 60438 Frankfurt am Main Germany; ^4^ Helmholtz Institut for Pharmaceutical Research Saarland (HIPS)/Helmholtz Center for Infection Research (HZI) Campus E8.1 66123 Saarbrücken Germany; ^5^ Department of Pharmacy Saarland University Campus E8.1 66123 Saarbrücken Germany; ^6^ German Centre for Infection Research (DZIF) Partner Site Hannover‐Braunschweig 66123 Saarbrücken Germany; ^7^ Institute of Biochemistry II Medical Faculty Goethe‐University Frankfurt am Main and Buchmann Institute for Molecular Life Sciences 60438 Frankfurt am Main Germany; ^8^ Frankfurt Cancer Institute Goethe University 60596 Frankfurt am Main Germany; ^9^ Fida Biosystems Aps Generatorvej 6 Soborg 2860 Denmark

**Keywords:** ligand screening, mass spectrometry, native mass spectrometry, proteomics, Taylor dispersion

## Abstract

Native mass spectrometry (MS) is an important technique in structural biology and drug discovery, due to its ability to study non‐covalent assemblies in the gas phase. Drawbacks include the incompatibility of electrospray ionization (ESI) with non‐volatile salts and the risk of protein signal suppression by small molecules. Overcoming these often requires offline buffer exchange and/or parallel sample preparation to other methods, reducing the adoption and throughput of native MS. Here, we exploit the dynamics of analytes flowing through an open tubular capillary to keep molecules with a small hydrodynamic radius (e.g., salts) inside a Taylor dispersion regime while pushing larger species (e.g., proteins) into a non‐Taylor regime. As such, larger species elute earlier, and are effectively buffer exchanged within the capillary in seconds. In addition to desalting of proteins injected in biologically relevant buffers we demonstrate separation of unbound small molecules from protein‐ligand complexes, enabling multiplexed ligand screening. Finally, we investigated the dependence of the critical flow rate for non‐Taylor behavior on protein size, enabling limited size‐based separation of proteins. Taylor/non‐Taylor dispersion mass spectrometry (TNT‐MS) was implemented using an unmodified liquid chromatography ‐ mass spectrometry (LC‐MS) system operated without a chromatographic column and coupled to an autosampler, which allowed significant automation.

## Graphical abstract

Native mass spectrometry enables the label‐free study of noncovalent protein‐ligand complexes; however, it is incompatible with conventional molecular biology buffers. By exploiting the dependence of flow dynamics on the hydrodynamic radius, proteins are seperated, including protein‐ligand complexes, from non‐volatile salts and unbound small molecules in an open tubular capillary, allowing rapid desalting and multiplexed ligand screening.

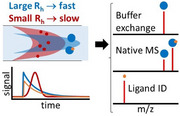

## 1 Introduction

The analysis of proteins in their native state, preserving non‐covalent interactions and higher‐order structure is an important topic in structural biology. Although conventional techniques (e.g., X‐ray crystallography or nuclear magnetic resonance) have been used successfully for many years, native mass spectrometry has recently emerged and offers unique advantages.^[^ ^1^ ^]^ Based on a mass over charge (*m/z*) readout, native MS allows for the direct observation of intact proteins and complexes. It can be used to obtain valuable information, including stoichiometry, conformation, and ligand binding.^[^ ^2^ ^]^ Native MS requires low amounts of the sample at concentrations typically in the micromolar range, and importantly, can analyze heterogeneous mixtures with several proteins and small molecules.^[^ ^1^ ^]^ However, native MS is not compatible with non‐volatile salts and is often time‐consuming due to the need for offline desalting or buffer exchange.^[^ ^3^ ^]^

Native MS has tremendous potential in drug discovery, which has led to a number of recent publications on this topic.^[^ ^4^, ^5^, ^6^, ^7^, ^8^, ^9^, ^10^, ^11^ ^]^ However, the lack of automation has to some extent limited the adoption of native MS outside its own scientific community. Moreover, the ionization of multicomponent samples (e.g., including protein, protein‐ligand complex, and several small‐molecule ligand candidates) simultaneously, is somewhat problematic in ESI. Smaller species often have a higher ionization efficiency, which can cause signal suppression of the proteins and complexes.^[^ ^12^, ^13^ ^]^ To address these challenges, there is a growing demand for automated, high‐throughput native MS methods.^[^ ^14^, ^15^ ^]^ Previous efforts have included the use of online buffer exchange with a short size‐exclusion chromatography (SEC) column, and the use of sub‐micron electrospray emitters, which seem to improve tolerance toward non‐volatile species.^[^ ^16^, ^17^ ^]^ Such developments can make native MS more accessible to other researchers, including molecular biologists and medicinal chemists.

Recently, flow‐induced dispersion analysis (FIDA), also known as Taylor–Aris dispersion analysis (TADA) has been explored for studying proteins and protein complexes.^[^ ^18^, ^19^, ^20^ ^]^ In brief, FIDA exploits the characteristic axial dispersion of solutes in a laminar flow according to their size/shape, due to the interplay between radial diffusion and axial convection in a Taylor dispersion regime.^[^ ^21^, ^22^ ^]^ This concept was recently applied by Buell and colleagues in the investigation of liquid–liquid phase separation.^[^ ^18^ ^]^ Gaspar and colleagues reported the adaptation of a capillary electrophoresis instrument, coupled to a mass spectrometer to automate protein desalting and buffer exchange.^[^ ^19^, ^20^ ^]^ In this work, the non‐volatile small molecules were concentrated in the center of the elution profile (where they strongly suppressed protein ionization) but as the protein was diluted across a longer stretch of the capillary, buffer‐exchanged protein signal could be detected in the early and late stages of the peak.

**Figure 1:**
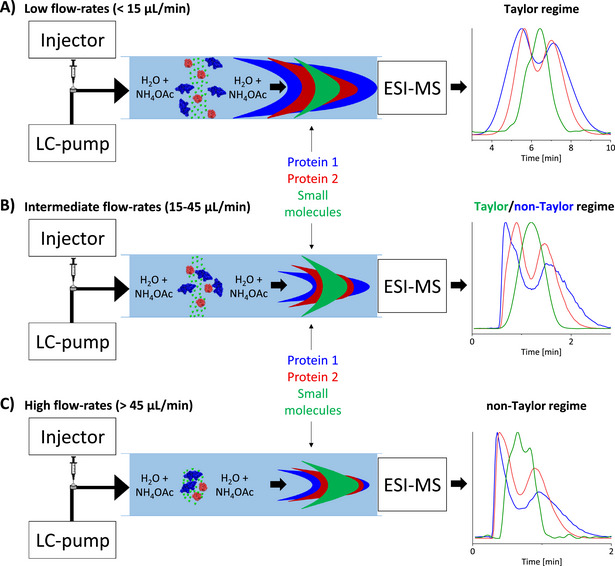
Schematic illustration of the dispersion regimes covered in this work according to the molecular mass. A) Taylor dispersion regime: all molecules diffuse radially toward the wall completely before leaving the capillary, resulting in Gaussian elution profiles with widths that depend on their hydrodynamic radius; B) At higher flow rates, larger molecules elute from the capillary before they complete their radial diffusion, while smaller ones have enough time to reach the capillary inner surface; and C) Non‐Taylor regime: Analytes remain in the center of the capillary at high flow rates, flowing through the capillary mostly under a convective regime. Note: The green trace represents small molecules, which would need significantly higher flow rates than are conveniently achievable to be pushed fully out of the Taylor regime, explaining why in (C) it shows considerable asymmetry but does not follow exactly the same pattern as the red (FKBP12, 12 kDa) and blue (bovine serum albumin, 66 kDa) traces. The data points used in these plots were acquired experimentally with TNT‐MS. Note that the “dip” in protein signal near the middle of the elution window is due to ion suppression, as small molecules and salts are concentrated in this range.

Here, we show a novel approach that exploits the ability to adjust the flow rate to selectively push analytes outside of the Taylor dispersion regime based on their hydrodynamic radius (see **Figure** **1**). We have named this method “Taylor/non‐Taylor dispersion mass spectrometry” (TNT‐MS). Compared to previous publications that used Taylor dispersion combined with MS,^[^ ^19^, ^20^ ^]^ our method has several key advantages. Compared to molecules in the Taylor dispersion regime, the elution maximum for non‐Taylor behavior is 50% faster (see Figure S1, Supporting Information). As will be discussed later, a higher flow rate is also required to induce non‐Taylor behavior. These two factors together mean that analysis of the same molecule, on the same instrument, with TNT‐MS is always faster than analysis in a Taylor regime, by a factor greater than two. This faster analysis also reduces dilution, which helps preserve labile interactions (see Figure S1, Supporting Information).

So far, only a few studies have explored the diffusion profile from mixtures of compounds under varied laminar flow regimes, and none have used MS coupling.^[^ ^23^, ^24^, ^25^ ^]^ These previous studies have shown that it is indeed possible to separate two compounds by merely passing them simultaneously through an open tubular capillary. The interplay between how long it takes for a compound to diffuse radially across the capillary, until it reaches the wall (time 1, t_1_), versus how long it takes to elute from the capillary (time 2, t_2_) is crucial. If the radial diffusion is complete before one compound elutes (t_1_ < t_2_), it is in a Taylor dispersion regime and its peak shape is described by a Gaussian distribution; conversely, if the radial diffusion of another compound is incomplete before it elutes (t_1_ > t_2_) under the same conditions, it is in a non‐Taylor regime, and its signal rapidly reaches a maximum (theoretically at half the time as the maximum of the Gaussian distribution of the Taylor regime) and then slowly decays (see Figure S1, Supporting Information). Taylor‐ or non‐Taylor conditions can be achieved by either selecting specific flow rates or capillary dimensions and are highly dependent on the properties (mainly the hydrodynamic radius) of each compound.^[^ ^23^, ^24^, ^25^ ^]^

Based on the above, a flow rate can be chosen so that small molecules, salts, and buffer components fall into the Taylor dispersion regime (Gaussian elution profile), while larger species such as proteins or complexes fall into the non‐Taylor regime. The Taylor‐ or non‐Taylor behavior of a specific compound can be predicted from parameters such as the Péclet number (Pe) – which describes the relative importance of mass transport by convection over diffusion – and tau (τ), which is a measure of the radial diffusion time. These parameters can be altered as a function of the flow rate.^[^ ^23^, ^24^, ^25^ ^]^ TNT‐MS allows rapid desalting (separation of non‐volatile small molecules and salts from macromolecules), ligand screening (separation of unbound ligand candidates from protein‐ligand complexes), and to some extent, even size‐based separation of proteins. Experiments typically took less than 3 min, with signals for the proteins of interest typically (depending on the flow rate used) obtained after around 30 s. Sample consumption was comparable to typical static nano‐ESI‐based native MS experiments. The method was implemented using a commercially available LC‐MS system operated without a chromatographic column. Compared to other separation techniques such as SEC and ion exchange chromatography (IEX),^[^ ^16^, ^26^, ^27^, ^28^, ^29^, ^30^ ^]^ TNT‐MS is very fast (less than 3 min) and does not use a stationary phase, which reduces the risk of carry‐over and/or destabilization of labile complexes. In addition, our method is very easy to implement and inexpensive, as only a pump and open tubular capillary are required. Compared to capillary electrophoresis,^[^ ^31^, ^32^ ^]^ our method has the advantage of using a simple setup and voltage‐free separation, with automatic desalting, making it highly compatible with ESI‐MS and suitable for fragile analytes.

We demonstrate online desalting and size‐based differences in elution behavior at different flow rates using model proteins spanning an extensive molecular mass range (12–149.5 kDa). As a proof‐of‐concept experiment for multiplexed ligand screening with native TNT‐MS, we used five pharmacologically relevant proteins: AP2‐associated protein kinase 1 (AAK1), baculoviral IAP repeat‐containing protein 4 (BIRC4), cereblon (CRBN), LIM domain kinase 1 (LIMK1), and serine/threonine kinase 17A (STK17A). AAK1 is a neuron‐ and liver‐specific protein crucial for clathrin‐mediated endocytosis.^[^ ^33^ ^]^ It is involved in many neurodegenerative diseases (e.g., schizophrenia, Parkinson's, and Alzheimer's), and viral infections (e.g., hepatitis C, dengue, and Ebola).^[^ ^34^ ^]^ BIRC4 is an apoptosis inhibitor that suppresses caspase activity. It plays a crucial role in cellular homeostasis and cancer progression.^[^ ^35^ ^]^ CRBN is a substrate adapter protein of the Cullin‐Ring ligase 4 complex, essential for ubiquitin‐mediated protein degradation in cells.^[^ ^36^ ^]^ It is the most used E3‐ligase receptor in the field of targeted protein degradation so far. Note that we used a recently developed CRBN construct here that is easier to handle and express in *E. coli* than the wild‐type protein.^[^ ^37^ ^]^ LIMK1 is a serine/threonine kinase that regulates the actin cytoskeleton and is very important in cell morphology, migration, and synaptic plasticity.^[^ ^38^ ^]^ Studies on LIMK1 have demonstrated that its overexpression might be related to cancer progression and metastasis.^[^ ^39^ ^]^ STK17A is another serine/threonine kinase related with cell cycle regulation, apoptosis, and acts in response to DNA damage, phosphorylating important substrates such as p53 and caspase 3.^[^ ^40^, ^41^ ^]^ STK17A is a member of the death‐associated protein family and therefore has been linked to several types of cancer.^[^ ^40^, ^41^, ^42^ ^]^ Each protein was incubated with a small library of 26 compounds including known ligands and subsequently introduced into the LC‐MS system with an autosampler, without any additional sample pre‐treatment or buffer exchange.

## 2 Results and Discussion

**Figure 2:**
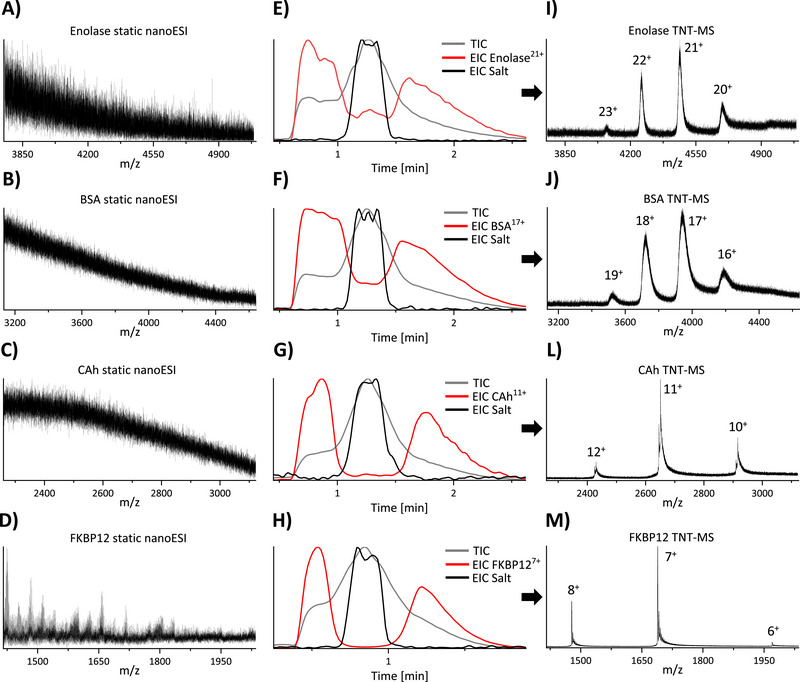
A–D) Static nano‐ESI‐MS of proteins dissolved in 200 mm NH_4_OAc and 25 mm NaCl, showing uninterpretable data. E–H) Total ion chromatograms (TICs) were acquired with TNT‐MS of the same samples, as well as protein and salt cluster EICs. There is a clear difference in EIC peak shape for proteins and salt clusters. I–M) Spectra obtained from TNT‐MS measurements by averaging the entire TIC shown in (E–H), highlighting the efficient desalting provided by this method. Note: TNT‐MS of enolase, BSA, and CAh was performed using a flow rate of 35 µL min^−1^; TNT‐MS of FKBP12 was performed at 50 µL min^−1^. Full‐*m/z* range spectra are shown in Figure S3A–D (Supporting Information).

As a control experiment, the model proteins enolase (93 kDa), bovine serum albumin (BSA, 66 kDa), carbonic anhydrase (CAh, 29 kDa), and FKBP12 (12 kDa) were dissolved in 200 mm aqueous ammonium acetate (NH_4_OAc) with 25 mm NaCl and sprayed using static nano‐ESI from glass emitters (**Figure** **2**A–D). As expected, no protein signal was detectable in the resulting spectra due to the strong ion suppression effect of NaCl. The same protein samples were next analyzed with TNT‐MS, and their elution profiles are shown in Figure 2E–H. These measurements were acquired under conditions chosen to push the proteins out of the Taylor dispersion regime while keeping small molecules inside. Hence, the extracted ion chromatograms (EICs) of the proteins reached their maximum intensity earlier compared to salt cluster EICs, resulting in a window where the protein was effectively desalted. Small molecules (e.g., salts) were concentrated in the middle of the elution profile, resulting in ion suppression in this range, and a second window (after the salt peak) where desalted protein can also be detected, albeit at a lower intensity than in the earlier window. Importantly, the MS spectra shown in Figure 2I–M were obtained by averaging signal over the entire time range of the experiment, including the central part (effectively averaging two regions of clean protein signal with one region containing mostly noise, resulting in acceptable quality overall). Fast online desalting can also be achieved for biologically relevant buffer systems, as we show in Figure S2 (Supporting Information). Here we measured CAh in 20 mm HEPES pH 7.5, 200 mm NaCl, 1 mm TCEP, and 5% glycerol with TNT‐MS and achieved excellent online desalting. For this example, we also show how the protein signal in the spectrum changes over time during the TNT‐MS measurement.

**Figure 3:**
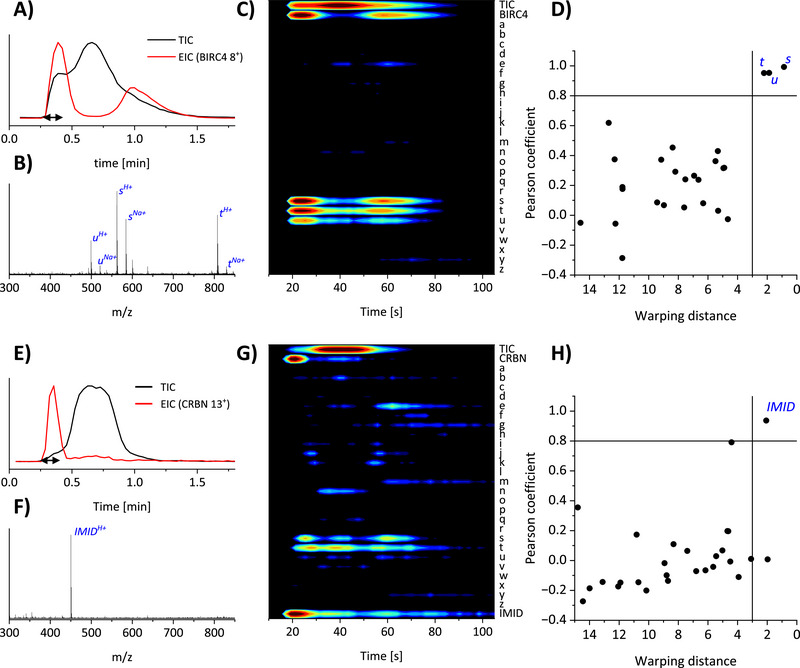
TNT‐MS ligand screening for BIRC4 and CRBN with a mixture of 26 small‐molecule compounds (+ iberdomide (IMID) for CRBN) at a flow rate of 60 µL min^−1^. Normalized TIC and EIC of the proteins A) BIRC4 and E) CRBN. Black arrows indicate the area selected to generate the corresponding low‐*m/z* spectra in (B,F). In these spectra, the corresponding ligand signals are clearly visible and labeled in blue. Contour plots for ligand screening with (C) BIRC4 and (G) CRBN show the intensity distributions (red = highest, blue = lowest) in the high‐energy channel for the TIC, EIC of all ligands (as [M+H]^+^ ions), as well as the EIC of the most intense charge state of each model protein. TICs and protein EICs were normalized individually, while small‐molecule EICs were normalized collectively for each measurement. The horizontal axis represents time. As a more quantitative analysis, the Pearson correlation coefficient of each small molecule EIC is shown together with its dynamic time‐warping distance for the proteins (D) BIRC4 and (H) CRBN. A warping distance of < 3 and Pearson coefficient of >0.80 were selected as threshold values to identify binding ligands. Additional ligand screening experiments for the kinases LIMK1, AAK1, and STK17A are shown in Figure S4 (Supporting Information). The EICs of the identified ligands along with the negative control compound n are shown in Figure S5 (Supporting Information). Low‐energy native spectra showing mass shifts due to ligand binding can be found in Figure S6 (Supporting Information).

Next, we used TNT‐MS for the identification of binding ligands to target proteins. In this approach, protein‐ligand complexes remain intact during ionization but can be subjected to collision‐induced dissociation (CID) in the gas phase, resulting in the ejection of the ligand, usually as a singly‐charged ion. The EIC of the ejected ligand (which can be detected with high mass accuracy in the low‐*m/z* range) and the protein should thus match; in contrast, non‐binding compounds are expected to stay in the Taylor dispersion regime and thus display significantly different elution profiles. For additional confidence, the acquisition of spectra without collisional activation during the same TNT‐MS experiment (done by continuously switching between a low and high voltage offset in the collision cell) allows direct observation of the native‐like protein‐ligand complexes. The average analysis time in these experiments was less than 2 min per sample, with the protein‐ligand complex usually being detected after less than 30 s (see **Figure** **3**; Figure S4, Supporting Information).

We used three data processing methods to identify ligands. The first method was to examine the low‐*m/z* range of the spectra acquired with high collision energy during the early protein elution window. This showed that for the protein BIRC4 (Figure 3A,B) the known ligands s, t, and u were clearly recognized. In addition, signals for the sodium adducts of the three ligands were detected. Besides the ligands, hardly any or only very weak signals were observed. For the CRBN ligand screening (Figure 3E,F), we observed one clearly distinct signal in the low‐*m/z* range, matching the known ligand iberdomide, while no other signals were observed (note: we only added this ligand to the compound library for the CRBN screening for safety reasons). For AAK1 (Figure S4B,E, Supporting Information) and STK17A (Figure S4C,F, Supporting Information), mainly the known ligands x and z were visible, although for STK17A further signals with comparatively low intensity were observed. For LIMK1 (Figure S4A,D, Supporting Information), the known ligand x stood out, while low‐intensity signals were observed for the known ligand v and for compound z. The latter result was surprising as compound z was not reported before as a binder for LIMK1, although it is known to bind many other kinases.^[^ ^43^ ^]^ As ligands v and z have a mass difference of only 1 Da, it was not possible to use the low‐energy native MS data to verify whether both compounds were binding to LIMK1 (Figure S6C, Supporting Information). We therefore performed an additional competition assay using TNT‐MS with a sample containing LIMK1, ligand x, a non‐binder (compound s), and compound z (Figure S7, Supporting Information). The native MS data clearly showed complexes of LIMK1 with both x and z, confirming the identification of the latter as a binding ligand by TNT‐MS. The signal intensity of the complex with ligand z was considerably lower than that of the complex with ligand x, reflecting the lower (and hence previously unreported) affinity of this interaction. TNT‐MS combined with this data processing method is thus clearly useful to rapidly identify ligands in an untargeted manner; however, one downside is that it depends on the accurate selection of the correct EIC region across which to average the MS signal.

Our second data analysis strategy for ligand identification was a targeted method. The EICs of all added small‐molecule compounds were compared to the protein EIC. For convenience, the EICs can be shown in a contour plot. The ligands s, t, u in the screening experiment with BIRC4 (Figure 3C) had a clearly similar elution profile to that of the protein and produced a high signal intensity, which was consistent with the signals in Figure 3B. Similarly, we can see for the ligand screening with the E3 ligase CRBN (Figure 3G) that the protein EIC was very similar to the EIC of iberdomide, while the intensity was also high in comparison to the other ligands a–z. The analyses with LIMK1 (Figure S4G, Supporting Information), AAK1 (Figure S4H, Supporting Information), and STK17A (Figure S4I, Supporting Information) also allowed the identification of binding ligands (including ligand z for LIMK1 as discovered in this work, cf. supra). This data analysis workflow is helpful to obtain a comprehensive picture; however, the reliance on visual inspection can result in overlooking weakly binding or poorly ionizing ligands, as these will only show up with low intensity and can be “overwhelmed” by stronger signals. Therefore, a third analysis was performed based on Pearson correlation coefficients and dynamic time warping (DTW).

Pearson correlation can be used to quantify the similarity between two chromatograms based on their signal patterns over time.^[^ ^44^ ^]^ This approach focuses specifically on the correlation of signal fluctuations and not on absolute intensities. A high Pearson correlation (> 0.80) indicates that the temporal profiles of two chromatograms show strong or even very strong similarity, which in our case suggests ligand binding.^[^ ^45^ ^]^ A complementary method for comparing chromatograms is DTW. DTW can be used to evaluate the structural similarity between chromatograms.^[^ ^46^ ^]^ While Pearson correlation is sensitive to minor differences in retention time, DTW allows for more flexible pattern recognition by accounting for local variations in the signal profiles by using a non‐linear correlation.^[^ ^47^ ^]^ To ensure that the DTW analysis is unaffected by absolute intensity differences, both ligand and protein EICs were normalized to a range of 0–1 prior to each calculation. This normalization step ensures that the focus of the analysis is on relative structural patterns of intensity profiles while avoiding unwanted temporal skewing.

The data presented in Figure 3D showed that the Pearson correlation and dynamic time warping (DTW) analyses produced results that were consistent with the first two data analysis methods, effectively distinguishing the three binding ligands s, t, and u from non‐binding controls in the ligand screening with BIRC4. For cereblon (Figure 3H), AAK1 (Figure S4K, Supporting Information), and STK17A (Figure S4L, Supporting Information), the method also identified the known ligands. Interestingly, for LIMK1 (Figure S4J, Supporting Information), the ligands v and x were clearly detected as a cluster for a positive binder. The weakly‐binding, previously unreported ligand z, on the other hand, showed a slightly lower warping distance, but has a relatively high Pearson coefficient and would therefore be classified as more likely to be a positive binder (as confirmed by native MS; cf. supra).

A major strength of the method using DTW and Pearson correlation lies in its ability to quantitatively detect positive binders regardless of their signal intensity and thus ionization. However, when analyzing weakly binding ligands, there is a risk that low signal‐to‐noise ratios lead to reduced Pearson correlation coefficients and increased DTW distances. For reliable identification of weakly binding ligands, we suggest setting the DTW and Pearson correlation thresholds to more tolerant values. False positives resulting from these analyses can then be effectively eliminated by a subsequent contour plot analysis. This multi‐step validation approach should ensure robust detection across a wider range of binding affinities. Another strength of using easily calculable parameters such as DTW and Pearson is that it could be automated in the future, allowing higher throughput without relying on (possibly subjective) visual inspection of contour plots.

The flow rates were optimized in order to exceed Taylor dispersion conditions for proteins while keeping small molecules in the Taylor regime. The transition between both regimes is primarily determined by two fundamental parameters: the Péclet number (*Pe*) and *τ*–given by Equations (1) and (2), respectively.^[^ ^23^, ^24^, ^25^ ^]^

(1) $$\Delta P=\frac{4\cdot Pe\cdot k_{B}\cdot T\cdot L}{3\cdot \pi \cdot {R_{c}}^{3}\cdot R_{h}}$$

(2) $$\Delta P=\frac{4\cdot k_{B}\cdot T\cdot L^{2}}{3\cdot \pi \cdot \tau \cdot r^{4}\cdot R_{h}}$$

where Δ*P* is the applied mobilizing pressure, *k_B_* is the Boltzmann constant, *T* is the absolute temperature, *L* is the capillary length, *R_c_* is the capillary radius, and *R_h_* is the hydrodynamic radius. Since the flow velocity can be set to a constant value using an LC pump, while the resulting pressure is adjusted, the Hagen‐Poiseuille Equation (3) can be used to write the formulas for *Pe* and *τ* in terms of flow velocity. This allows for the simplification of these equations into the following Equations (4) and (5):

(3) $$\Delta P=\frac{8\cdot \eta \cdot L\cdot Q}{\pi \cdot {R_{c}}^{4}}$$

(4) $$Pe=\frac{6\cdot \eta \cdot Q\cdot R_{h}}{k_{B}\cdot T\cdot R_{c}}$$

(5) $$\tau =\frac{k_{B}\cdot T\cdot L}{6\cdot \eta \cdot Q\cdot R_{h}}$$

where *η* is the solvent dynamic viscosity and *Q* is the flow rate. Through these relationships, it can be demonstrated that the critical Péclet number (*Pe* > 40) is always met for analytes with typical protein hydrodynamic radii at all flow rates we used in this work. Therefore, for our application, only τ is crucial in determining whether a given molecule falls within or outside of Taylor conditions, with τ < 0.37 being considered a clear indication of a non‐Taylor dispersion regime. Based on the definition of τ and our experimental setup, a key advantage is that the capillary internal diameter becomes irrelevant. The capillary length (91 cm in our experiments) and flow rate are thus the only crucial factors that determine whether the appropriate τ value is achieved for a protein with a specific hydrodynamic radius. Equation (5) shows that a shortening of the capillary results in smaller τ values, as the molecules have less time to undergo radial diffusion. Accordingly, for a particle with a given hydrodynamic radius, the flow rate must be increased to induce non‐Taylor behavior in longer capillaries. Furthermore, Equation (5) also shows that for a given capillary length, a particle with a larger *R_h_* will transition to a non‐Taylor dispersion regime at a lower flow rate than a smaller one.

**Figure 4:**
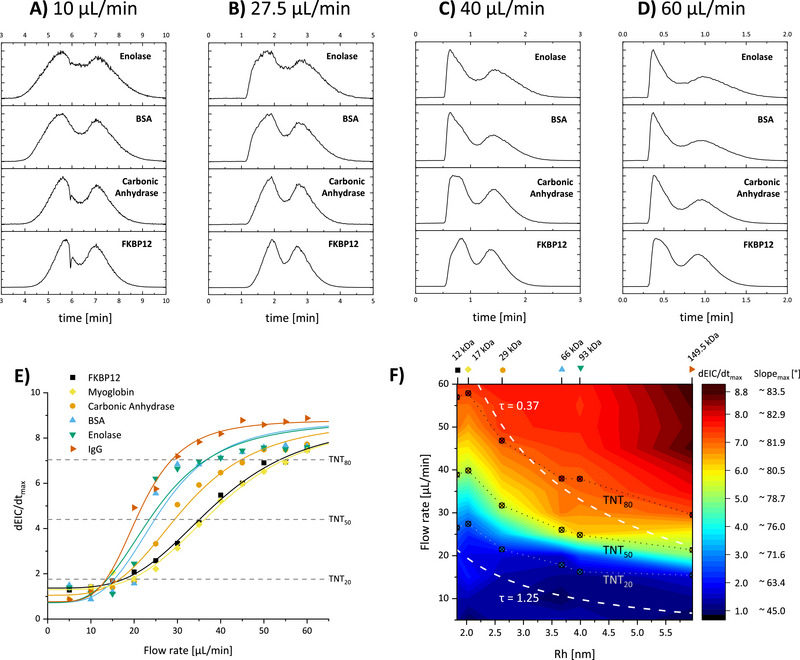
TNT‐MS of proteins of different sizes to study the transition between Taylor and non‐Taylor behavior. A–D) Protein EICs of FKBP12, carbonic anhydrase, BSA monomer, and enolase dimer at flow rates of (A) 10 µL min^−1^, (B) 27.5 µL min^−1^, (C) 40 µL min^−1^, and (D) 60 µL min^−1^. E) Maximum slope of the extracted ion chromatograms (dEIC/dt_max_) for FKBP12, myoglobin, carbonic anhydrase, BSA monomer, enolase dimer, and IgG at flow rates between 5 and 60 µL min^−1^. TNT_20_, TNT_50_, and TNT_80_ represent the effective flow rates at which 20%, 50%, and 80% of the maximum slope are reached. F) Contour plot of dEIC/dt_max_ as a function of flow rate and hydrodynamic radius based on the six proteins shown in (E), illustrating the transition from low (blue) to high (red) slope. TNT_20_, TNT_50_, and TNT_80_ values from (E) are overlaid, along with literature‐defined^[^ ^24^, ^25^ ^]^ critical τ values of 0.37 and 1.25. A full spectrum for the protein mix is shown in Figure S8D (Supporting Information) and for IgG1 and myoglobin in Figure S3E,F (Supporting Information).

In addition to rapid desalting (Figure 2) and ligand screening (Figure 3), TNT‐MS can be used to distinguish different proteins based on their size by carefully selecting the flow rate (**Figure** **4**). We analyzed a mixture of the proteins FKBP12, carbonic anhydrase, BSA, and enolase at flow rates between 5 and 60 µL min^−1^ (Figure 4A–D). When examining the respective EICs of the protein signals at a low flow rate of 10 µL min^−1^ (Figure 4A), all proteins show a Gaussian elution profile and are thus within the Taylor regime. As in previous figures, there is an intensity dip in the middle part of the elution window due to ion suppression by non‐volatile salts. With an increase of the flow rate to 27.5 µL min^−1^ (Figure 4B), the larger proteins enolase and BSA show a transition mode between Taylor‐ and non‐Taylor conditions, while the smaller proteins FKBP12 and carbonic anhydrase still show a Gaussian profile. By further increasing the flow rate to 40 µL min^−1^ (Figure 4C) enolase, BSA, and carbonic anhydrase show a typical non‐Taylor EIC, and at 60 µL min^−1^ (Figure 4D), FKBP12 also drops out of the Taylor regime. Interestingly, at a flow rate of 27.5 µL min^−1^, we can separate large (enolase and BSA) from small (FKBP12) proteins in the early region of the EICs, effectively performing low‐resolution ’columnless’ size‐based protein separation based on Taylor or non‐Taylor behavior (see Figure S8, Supporting Information).

To more quantitatively determine when a protein is inside or outside the Taylor regime, we calculated the first derivative of the normalized EICs and plotted the maximum slope (dEIC/dt_max_) against the flow velocity (Figure 4E), resulting in sigmoidal profiles. We will refer to the flow rate at the midpoint of these profiles as TNT_50_ values, and these correspond to the intermediate phase, where a protein transitions between both regimes. Similarly, TNT_20_ and TNT_80_ values can be defined and used as thresholds to ensure a protein shows clear Taylor or non‐Taylor behavior, respectively. For this analysis, we used the measured first derivates of all proteins of our protein mix consisting of FKBP12, carbonic anhydrase, BSA, and enolase, as well as from additional measurements with myoglobin (17 kDa) and IgG (149.5 kDa). Since τ defines whether a protein is inside or outside the Taylor dispersion regime, a higher flow rate is required to push smaller proteins out of the Taylor regime (as seen in Figure 4A–D). This trend is reflected in the calculated TNT_50_ values in Figure 4F, where we plotted the curves from Figure 4E in a heatmap visualization. The observed TNT_50_ values demonstrate that the transition point is centered between the critical literature τ values of 1.25 and 0.37.^[^ ^25^, ^48^ ^]^ The first value (1.25) was estimated by Cottet and colleagues,^[^ ^48^ ^]^ based on the condition that the average elution time for a molecule in a Taylor dispersion regime must be much longer than the time needed for it to diffuse in the capillary cross‐section, as pointed out by Taylor in his original work.^[^ ^49^ ^]^ Likewise, Okada and colleagues^[^ ^24^ ^]^ have experimentally demonstrated that a molecule is clearly outside Taylor dispersion conditions if τ < 0.37. The plot in Figure 4F can be used to estimate an appropriate flow rate for TNT‐MS experiments as a function of protein mass in the 12–149.5 kDa range.

## 3 Conclusion

TNT‐MS is a novel approach that allows high‐throughput analysis of native‐like proteins, including automated buffer exchange and ligand screening. Typical experiments took roughly 1.5 min. Since the method runs under isocratic conditions with aqueous ammonium acetate, there is no need for a conditioning step before injections, which provides an advantage for increasing the analytical throughput. An additional minute of washing between measurements might be useful in some cases, although no sign of carry‐over was spotted in our experiments. Furthermore, we carried out our experiments using an unmodified commercial LC‐MS system equipped with an autosampler, allowing for overnight measurements. Assuming 2.5 min per run (including a 1‐min washing step), 576 measurements could therefore be performed in 24 h, meaning that ≈15000 ligand candidates could be screened daily if–as in our work–each sample contains 26 ligands (although we note that we were limited to 26 compounds due to availability of drug‐like small molecules and there is no reason to assume this number represents a fundamental limit for the TNT‐MS method). Another advantage is that samples can be incubated in standard buffers for molecular biology and no offline buffer exchange into ammonium acetate solution is required. The time proteins spend in aqueous ammonium acetate is also significantly shorter compared to offline buffer exchange, which can be beneficial as ammonium acetate has very little buffering capacity at physiological pH and its pH tends to drop slightly over time due to decomposition and evaporation of NH_3_. Since TNT‐MS requires only an LC pump, an autosampler (although manual injection can be used as well), and a mass spectrometer, we believe TNT‐MS will be extensively adopted in many laboratories and represents important progress toward fully automated and democratized native MS.

## Conflict of Interest

The authors declare no conflict of interest.

## Supporting information



Supporting Information

## Data Availability

The data that support the findings of this study are available from the corresponding author upon reasonable request.
