# Supplementary material for: Native Taylor/Non‐Taylor Dispersion–Mass Spectrometry (TNT‐MS) Allows Rapid Protein Desalting and Multiplexed, Label‐Free Ligand Screening
Source: Small Methods. 2025 May 7;10(2):2500658. doi: 10.1002/smtd.202500658 (PMC12825326; doi:10.1002/smtd.202500658)
Supplement: Supplementary file 1 — Supporting Information [file SMTD-10-2500658-s001.pdf]

Supporting Information for: Native Taylor/Non-Taylor Dispersion – Mass Spectrometry (TNT-MS) Allows Rapid Protein Desalting and Multiplexed, Label-Free Ligand Screening

Jonathan Eisert,[1†] Edvaldo Vasconcelos Soares Maciel,[1†], Verena Dederer,[2,3] Aylin Berwanger,[4,5,6] Henry J. Bailey[7], Ivan Đikić[7], Stefan Knapp,[2,3,8] Martin Empting,[4,5,6] Sebastian Mathea,[2,3] Henrik Jensen,[9] Frederik Lermyte[1]*

[1]*Department of Chemistry, Clemens-Schöpf-Institute of Chemistry and Biochemistry, Technical University of Darmstadt, Peter-Grünberg-Strasse 4, 64287 Darmstadt, Germany*

[2]*Institute of Pharmaceutical Chemistry, Goethe University, Max-von-Laue-Str. 9, 60438 Frankfurt am Main, Germany*

[3]*Structural Genomics Consortium (SGC), Buchmann Institute for Life Sciences, Max-von-Laue-Str. 15, 60438 Frankfurt am Main, Germany*

[4]*Helmholtz Institut for Pharmaceutical Research Saarland (HIPS) / Helmholtz Center for Infection Research (HZI), Campus E8 1, 66123 Saarbrücken*

[5]Saarland University, Department of Pharmacy, Campus E8.1, 66123 Saarbrücken, Germany.

[6]German Centre for Infection Research (DZIF), Partner Site Hannover-Braunschweig, 66123 Saarbrücken, Germany

[7]*Institute of Biochemistry II, Medical Faculty, Goethe-University, Frankfurt am Main and Buchmann Institute for Molecular Life Sciences, Frankfurt am Main, Germany*

[8]*Frankfurt Cancer Institute, Goethe University, Frankfurt am Main, Germany*

[9]*Fida Biosystems Aps, Generatorvej 6, 2860 Soborg, Denmark*

[†]These authors contributed equally

*Correspondence: frederik.lermyte@tu-darmstadt.de

Experimental Section

TNT-MS measurements were performed with a Waters Acquity M-Class UPLC system (operated without an LC column) coupled to a Synapt XS ion mobility mass spectrometer (Waters, Wilmslow, UK). The MS was equipped with a low-flow ESI source operating in positive ion mode. Samples were injected into the system through a CombiPAL liquid autosampler (CTC Analytics AG, Zwingen, CH). The samples were injected as a plug into a mobile phase composed of 10 mM aqueous NH_4OAc (selected due to its compatibility with ESI [1,2]), flowing through an empty PEEK capillary (254 µM I.D. x 91 cm). PEEK capillaries offer a bioinert environment and avoid non-specific adsorption and the need for any pre-coating steps, which are often required for other tubing materials such as fused-silica or stainless steel.[3] Furthermore, PEEK has satisfactory mechanical strength and chemical resistance, and is considered suitable for LC-based applications under low- to moderate-pressures (up to ca. 440 bar).[4] Flow rates varied from 5-60 µL/min depending on the experiment as noted in the text and figure captions. Our setup consisted of an LC pump coupled to the PAL autosampler. After the injection loop, the 91 cm long, 254 µm inner diameter PEEK capillary was directly coupled to the low-flow source from Waters installed on the Synapt XS. The low-flow source had a 23.5 cm long, 50 µm inner diameter capillary. Thus, the combined capillary length we used was 114.5 cm. We conditioned our capillary with the running buffer (10 mM AmAc) at the specified flow rates. Using the autosampler, 2 µL of the sample was injected into the flow as a plug via a 2 µL loop. This sample was then transported with the flow towards the low-flow ESI source.

The main ESI parameters were set as follows: capillary voltage, 3.0 kV; sampling cone, 40 V; source temperature, 45 °C; desolvation temperature, 200 °C; desolvation gas flow, 550 L/h; and cone gas flow, 150 L/h. Native MS data were acquired under soft conditions, with a collision energy of 7 V in both the Trap and Transfer cell. In ligand screening experiments, the Trap cell was continuously alternated at a frequency of 0.5 Hz between low- and high-energy conditions (7 and 32 V, respectively). This method allows the detection of protein-ligand complexes in the low-energy data, and the ejected ligand(s) in the high-energy data. Further MS parameters are listed in **Table S1** in the **Supporting Information**. Data acquisition and analysis were carried out with MassLynx 4.2 (Waters, Wilmslow, UK), Excel (Microsoft, Redmond, USA), and Origin (OriginLab, Northampton, USA). EICs were smoothed (2x, 8 channels, Savitzky-Golay) for $dEIC/dt_{max}$ calculations to avoid spikes caused by noise. Hydrodynamic radii shown in **Figure 4** were measured with a Fida 1 instrument from Fida Biosystems ApS (Copenhagen, Denmark).

LC-grade water, ammonium acetate, and the proteins BSA (catalogue number AV7030), carbonic anhydrase (C2624), enolase (E6126), and myoglobin (M0630) were purchased from Merck (Darmstadt, Germany). IgG1 (catalogue number 186006552) was acquired from Waters. Information about expression of AAK1, BIRC4, FKBP12, LIMK1, STK17A, and CRBN can be found elsewhere.[5–8] The purchased proteins BSA, carbonic anhydrase, enolase, myoglobin, and IgG1 were dissolved in 200 mM AmAc without desalting. The proteins AAK1 (70 µM), BIRC4 (230 µM), LIMK1 (70 µM), and STK17A (80 µM) were stored in 20 mM HEPES pH 7.4, 150 mM NaCl, 2.5 mM $MgCl_2$, 0.5 mM TCEP, and 5% glycerol. CRBN (65 µM) was stored in 20 mM HEPES pH 7.5, 200 mM NaCl, 1 mM TCEP, and 5 % glycerol. FKBP12 (2.3 mM) was stored in 20 mM HEPES pH 8.0, and 20 mM NaCl. All subsequent dilution steps were done in 200 mM AmAc.

For the desalting experiments, proteins were diluted to a concentration of 15 µM in 25 mM NaCl or 20 mM HEPES pH 7.5, 200 mM NaCl, 1 mM TCEP, and 5 % glycerol, as described. For the ligand screening experiments, known ligands for CRBN, BIRC4, LIMK1, STK17A, and AAK1, as well as negative controls were sourced from in-house libraries of the Institute of Pharmaceutical Chemistry (Goethe University Frankfurt), the Buchmann Institute for Molecular Life Sciences (Goethe University Frankfurt), and the Helmholtz Institute for Pharmaceutical Research Saarland. The resulting curated batch of drug-like molecules had reasonable solubility (> 50 µM) in water, non-overlapping molecular masses, and

structural diversity. We first prepared a solution containing all 26 small-molecule compounds (listed in **Supporting Information Table S2**). The concentration of each ligand in the mix was 500 µM in 100% DMSO. This solution was subsequently mixed with the protein solution at a measuring concentration of 10 µM of each small molecule, and 25 µM of protein. The final DMSO concentration in the sample injected into the PEEK capillary was 2%. Approximately 8 µL of the resulting solution was consumed per measurement, which is comparable to typical static nano-ESI experiments with glass emitters, which also often use a similar concentration range. Note that only 2 µL was effectively injected into the capillary; the slightly larger sample volume required per measurement was necessary to fill the tubing length between the injection port and injection loop and this could be reduced with optimisation of the equipment. As such, TNT-MS has a similar sample consumption to typical native MS, especially if potential losses during offline buffer exchange are considered. The plug volume is a parameter that in principle can be varied; however, this is not expected to lead to significant differences in the Taylor dispersion behaviour. The main risk to avoid would be overfilling a capillary with a small internal diameter, which could lead to suboptimal performance.

For the Fida 1 experiments, protein samples were prepared at a concentration of 100 µM in 200 mM ammonium acetate (AmAc) solution. Each sample was analysed individually to measure intrinsic fluorescence. 200 mM AmAc buffer served as analyte buffer throughout all FIDA measurements. Hydrodynamic radius calculation was done using FIDA software V3.0 from Fida Biosystems ApS.

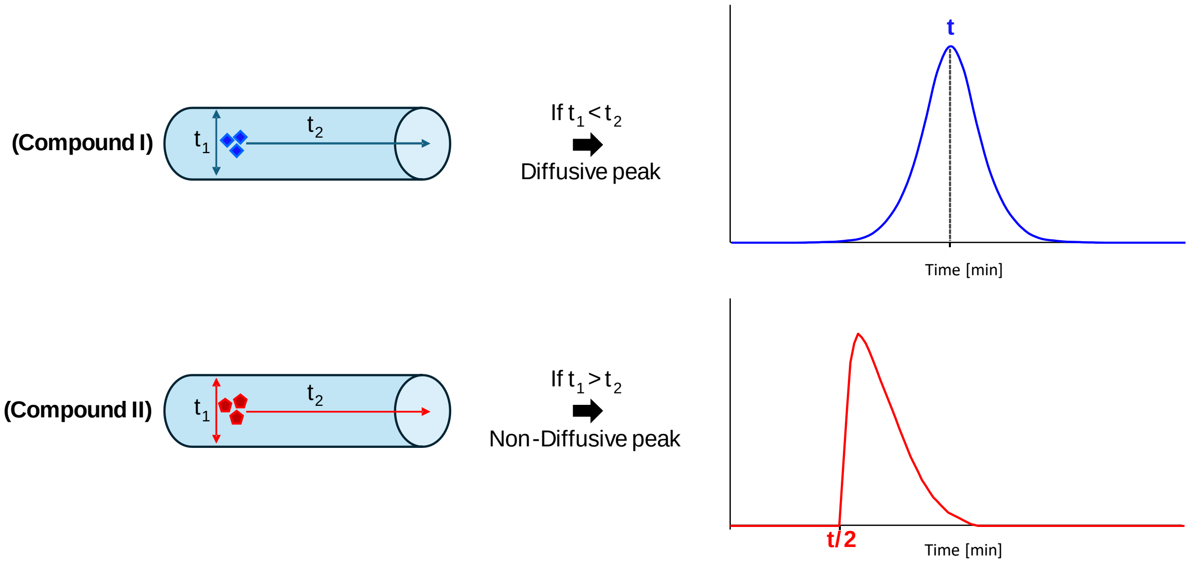

Figure S1. Peak profiles of two different compounds as a function of radial and axial diffusion under different laminar flow regimes: (I) Diffusive peak or Gaussian distribution, and (II) non-diffusive peak. The transition point between both regimes is distinct for every molecule and depends primarily on its hydrodynamic radius.

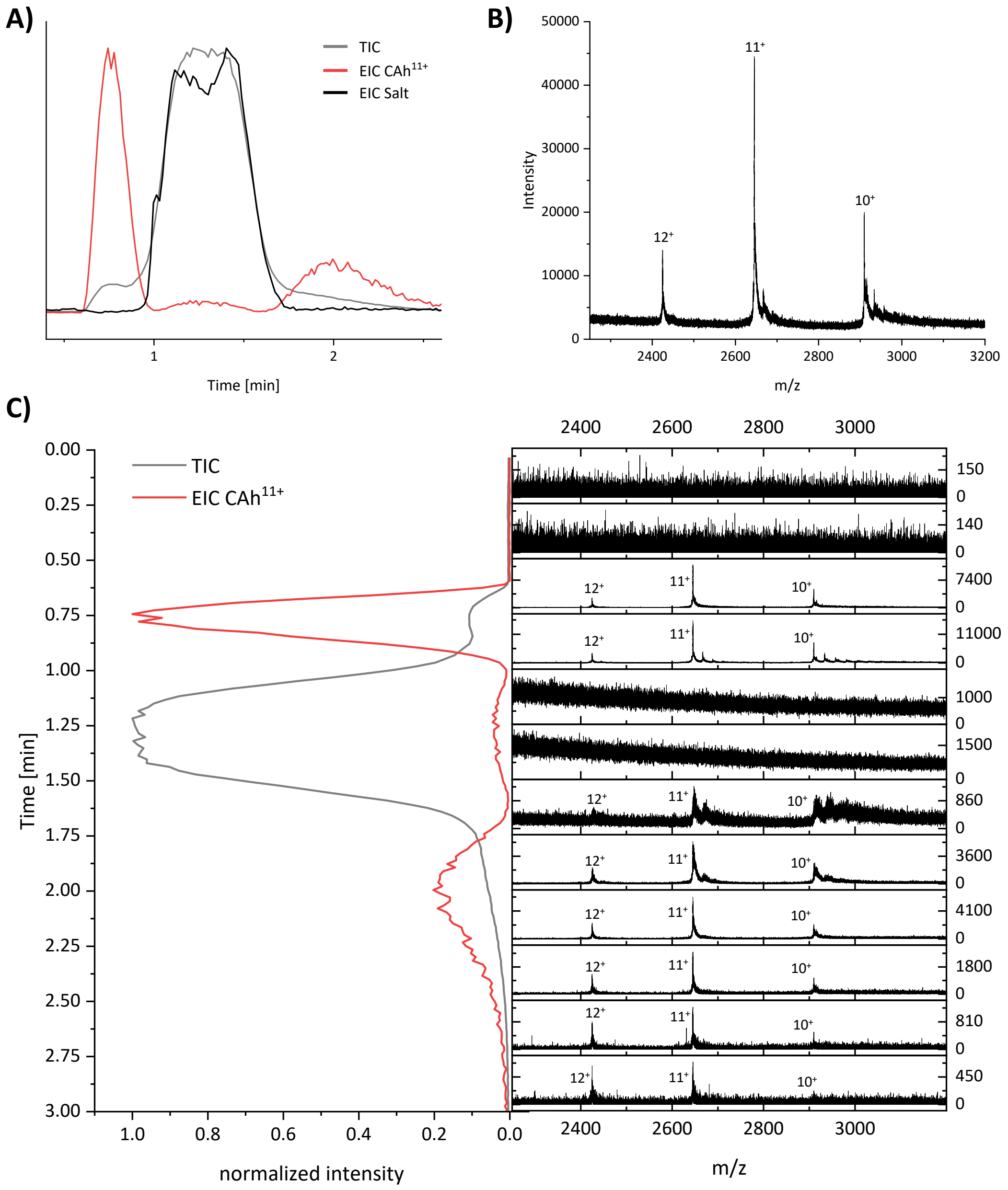

Fig S2. TNT-MS of carbonic anhydrase dissolved in 20 mM HEPES (pH 7.5), with 200 mM NaCl, 1 mM TCEP, and 5% glycerol without offline desalting. **(A)** Normalized TIC, protein EIC, and salt cluster EIC. **(B)** Spectrum obtained from TNT-measurements by summing spectra across the entire elution time shown in (A), demonstrating desalting efficiency in biologically relevant buffer systems. **(C)** Change in the spectrum from the TNT-MS measurement at different times. Each window (right) shows the summed spectrum of 25 seconds corresponding to the chromatogram on the left.

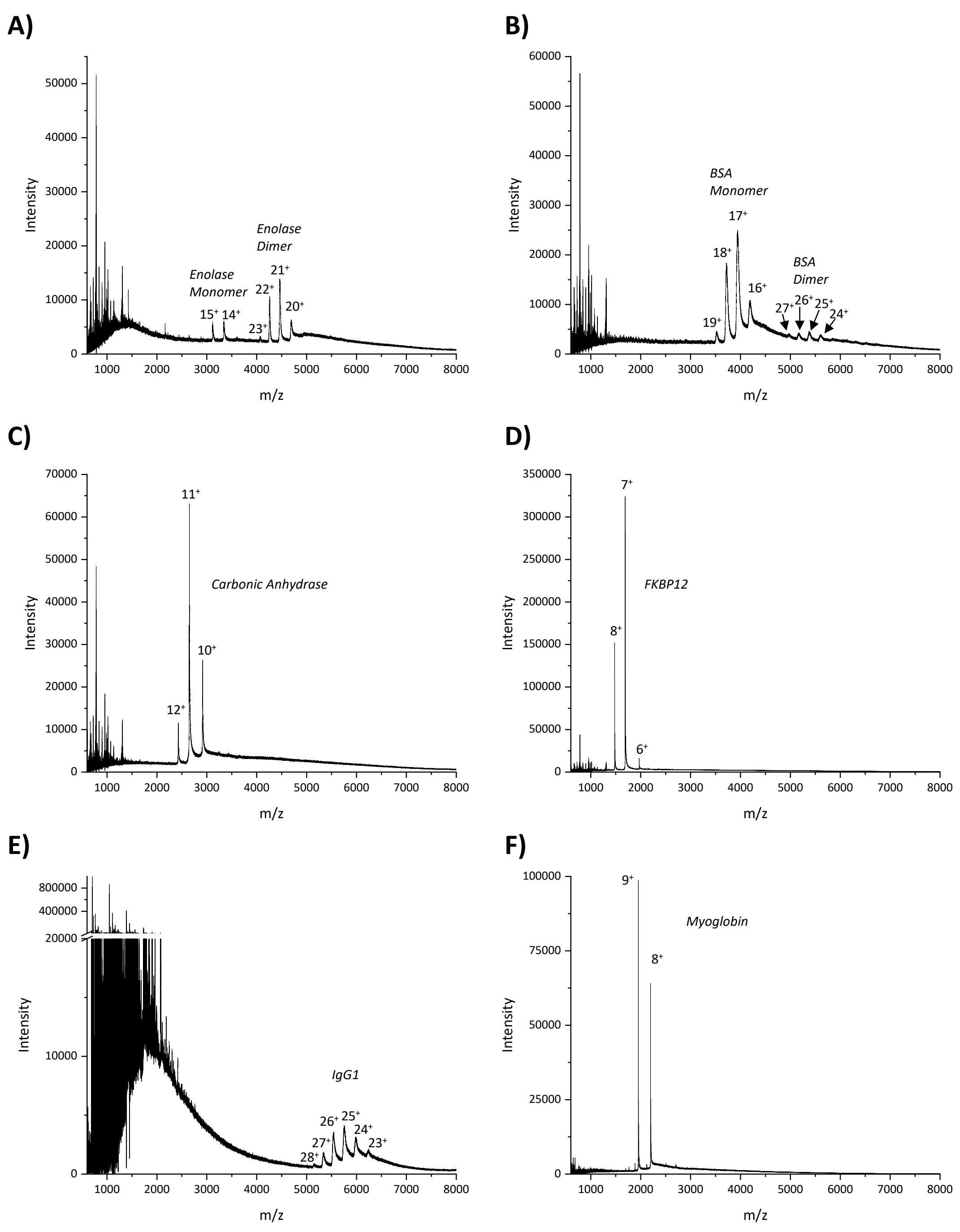

Fig S3. Full-*m/z* range spectra of TNT-MS experiments. Spectra show the protein desalting capabilities using TNT-MS for (**A)** enolase, **(B)** BSA, **(C)** carbonic anhydrase, and **(D)** FKBP12. The spectra are derived from the TNT-MS measurements from main text Figure 2. The spectra from TNT-MS measurements at 60 µL/min for **(E)** IgG1 (note: intensity axis break is due to very intense salt cluster signals) and **(F)** myoglobin are from the experiments shown in main text Figure 4. A full-*m/z* range spectrum of a mixture of FKBP12, carbonic anhydrase, BSA, and enolase is shown in Figure S8. The expected and measured molecular weights were 93.6 and 93.7 kDa for the enolase dimer, 66.4 and 66.9 kDa for the BSA monomer, 29.0 and 29.1 kDa for carbonic anhydrase, 17.6 and 17.5 kDa for myoglobin, 11.8 and 11.8 kDa for FKBP12, and 148.8 kDa and 149.5 kDa for the IgG standard.

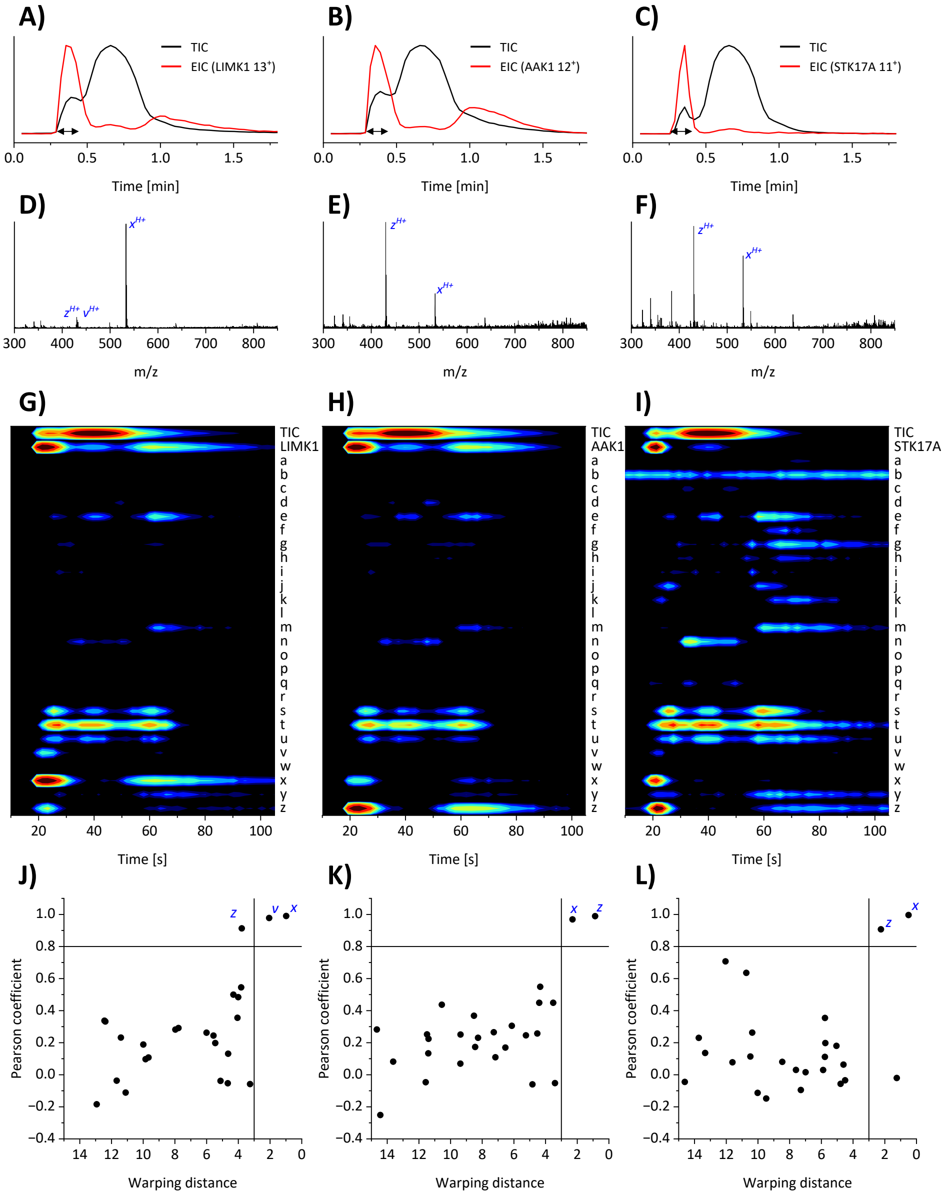

Figure S4. Ligand screening for the kinases LIMK1, AAK1, and STK17A with a mixture of 26 small-molecule compounds at a flow rate of 60 µL/min. Normalised TIC and EIC of the proteins **(A)** LIMK1, **(B)** AAK1, and **(C)** STK17A. Black arrows indicate the area selected to generate the respective low-*m/z* spectra shown in **(D)**, **(E)**, and **(F)**. In these spectra, the corresponding ligand signals are clearly visible and labelled in blue. Contour plots for ligand screening with **(G)** LIMK1, **(H)** AAK1, and **(I)** STK17A show the intensity distribution (red = highest, blue = lowest) in the high-energy channel for the TIC, EIC of all ligands (as $[M+H]^+$ ions), as well as the EIC of the most intense charge state of each model protein. TICs and protein EICs were normalised individually, while small-molecule EICs were normalised collectively for each measurement. The horizontal axis represents time. As a more quantitative analysis, the Pearson correlation coefficient of each small molecule EIC is shown together with its dynamic time warping distance for the proteins **(J)** LIMK1, **(K)** AAK1, and **(L)** STK17A. A warping distance of <3 and Pearson coefficient of >0.8 were selected as threshold values to identify binding ligands. The EICs of the identified ligands along with those of the proteins and of the negative control compound n are shown in **Supporting Information Fig. S5.** Low-energy native spectra showing the mass shift due to ligand binding can be found in **Supporting Information Fig. S6**.

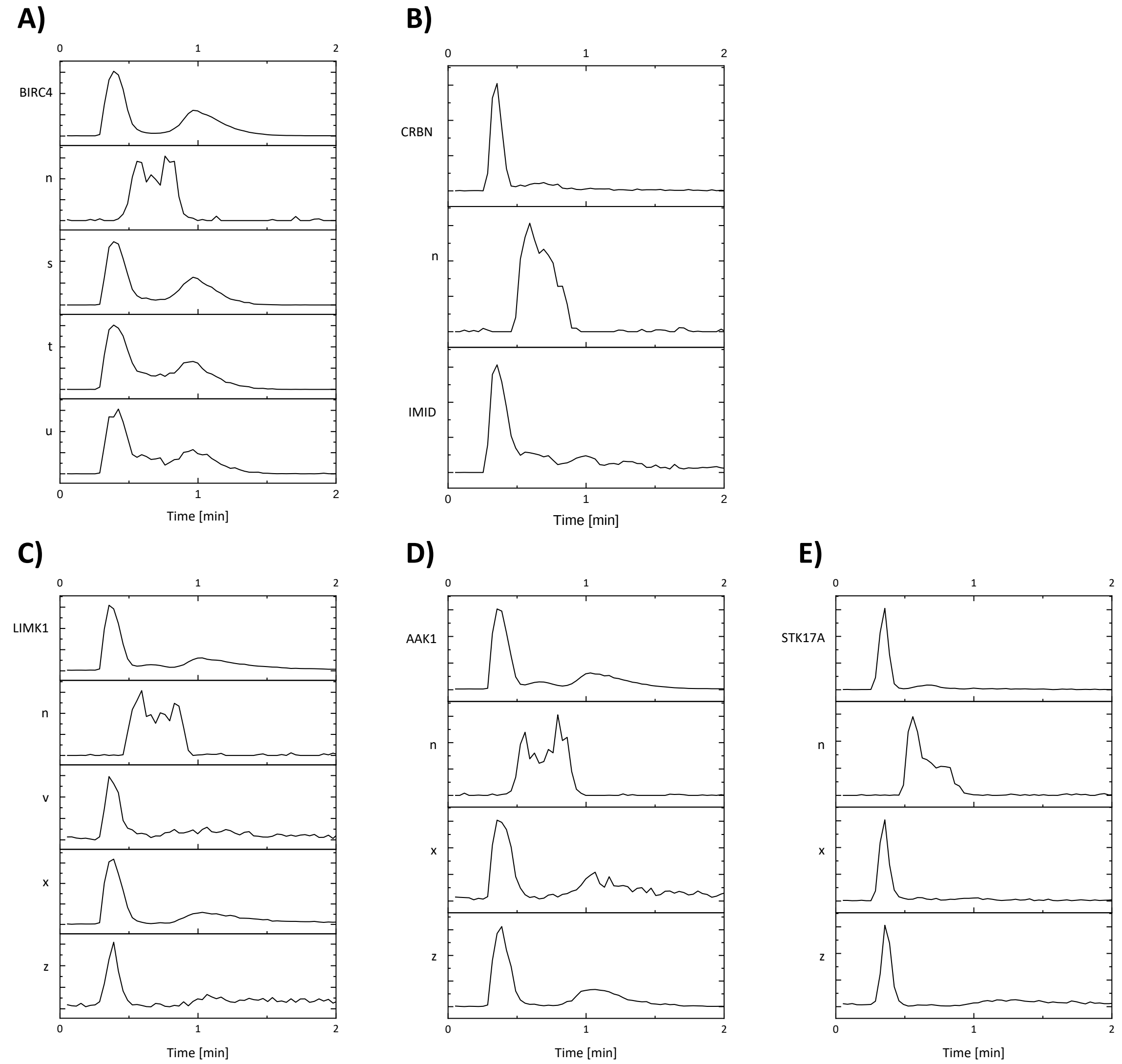

Figure S5. EIC comparison between proteins, identified ligands, and negative control (compound n) for **(A)** BIRC4, **(B)** CRBN, **(C)** LIMK1, **(D)** AAK1, and **(E)** STK17A.

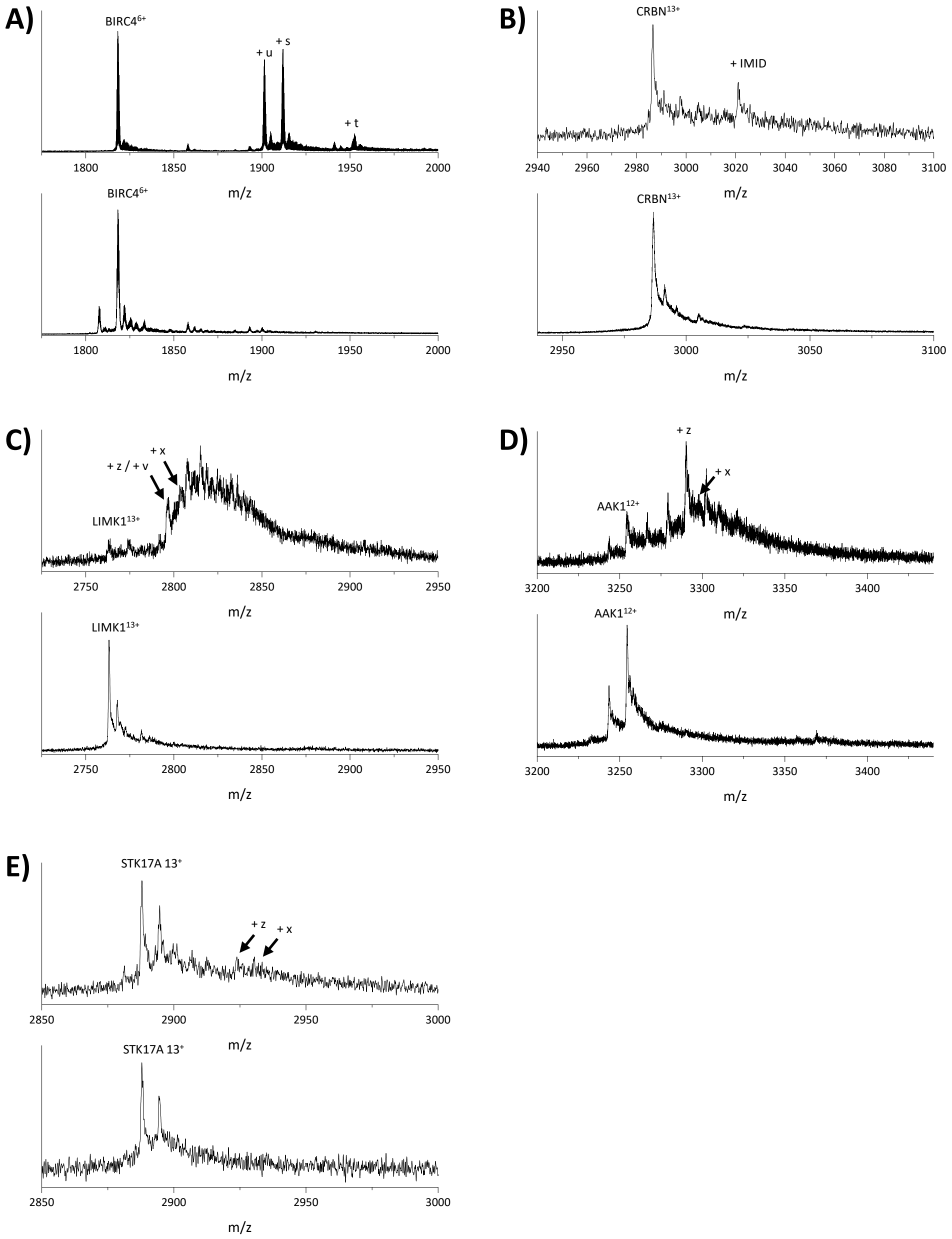

Figure S6. Native TNT-MS protein spectra of ligand screening experiments for **(A)** BIRC4, **(B)** CRBN, **(C)** LIMK1, **(D)** AAK1, and **(E)** STK17A. Top spectra in each panel show the protein-ligand complex(es) in the low-energy channel from the experiments of which the high-energy data are shown in **Fig. 3** and **Fig. S2**. Bottom spectra in each panel show control experiments of proteins without ligands added. Expected and measured molecular masses were 10.8 and 10.9 kDa for BIRC4, 38.7 and 38.8 kDa for CRBN, 36.0 and 35.9 kDa for LIMK1, 38.9and 39.0 kDa for AAK1, and 34.5 kDa and 34.6 kDa for STK17A.

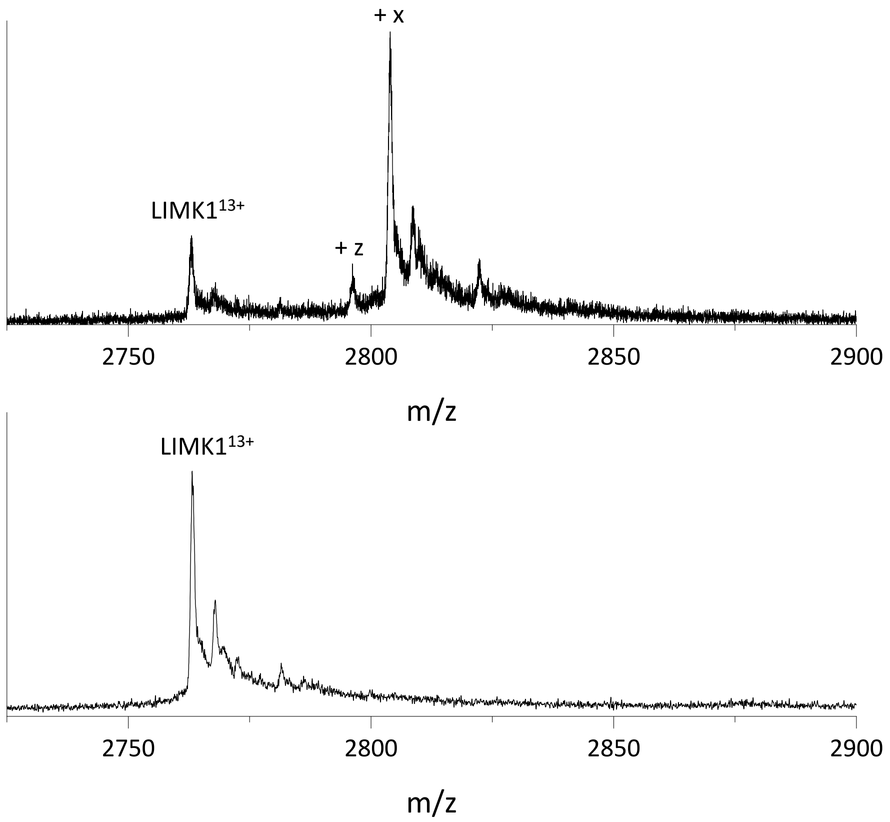

Figure S7. Native TNT-MS competition assay with LIMK1. Top spectrum shows mixture of LIMK1, positive control ligand x, suspected binding ligand z, and negative control compound s in a molar ratio of 2.5:1:1:1, respectively. Bottom spectrum shows a TNT-MS control spectrum of LIMK1 without added ligands.

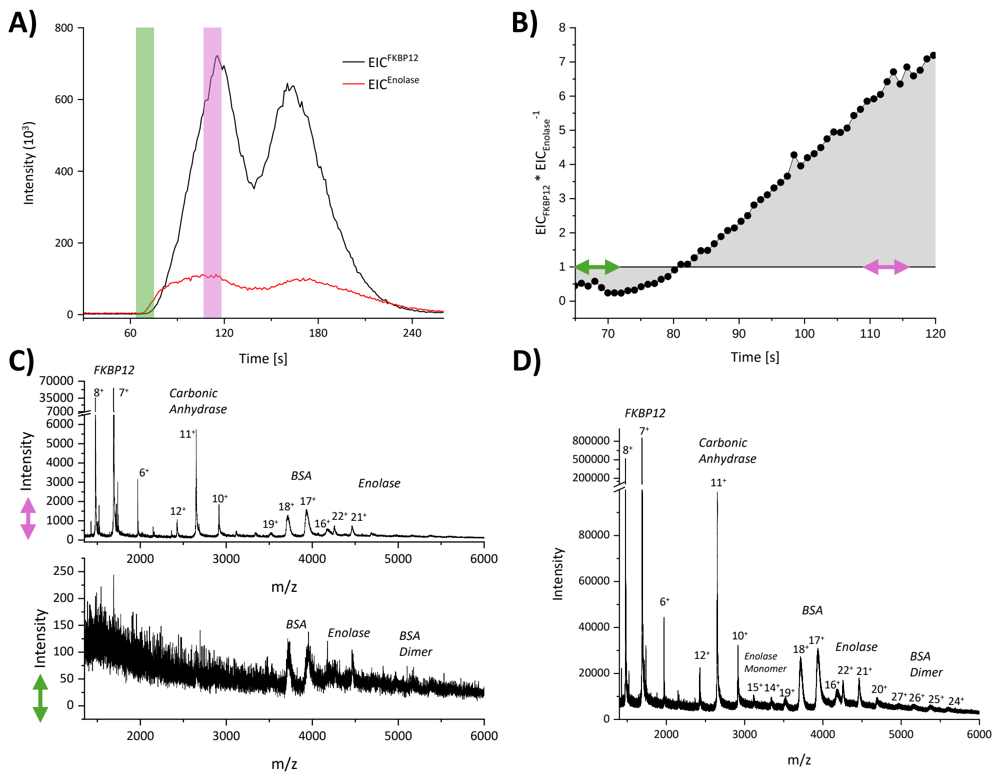

Figure S8. TNT-MS separation of different sized proteins (FKBP12, carbonic anhydrase, BSA, and enolase) at 27.5 µL/min (see **Fig. 4B** in the main text). **(A)** Overlay of the EICs of the most intense charge states of FKBP12 (7+) and dimeric enolase (21+). **(B)** Ratio of the intensities of the EICs of FKBP12 (7+) and dimeric enolase (21+) from (A) as a function of time. Values lower than 1 indicate a region of higher signal intensity of enolase compared to FKBP12. Values higher than 1 indicate a region of higher signal intensity of FKBP12 compared to enolase. Coloured arrows indicate the regions (same as those marked with semi-transparent rectangles in (A)) across which signal was averaged to generate the spectra shown in **(C)**, where a strong biasing toward larger or smaller proteins can be observed depending on the time window used, indicating a low-resolution 'columnless' size-based separation between different proteins. **(D)** Spectra of the protein mix summed over the complete TIC region.

	Native MS	Ligand screening	
		Low-energy	High-energy
**Capillary voltage (kV)**	3.0	3.2	3.2
**Sampling cone (V)**	12	12-40	12-40
**Source offset (V)**	0	1	1
**Source temperature (°C)**	30	30-45	30-45
**Desolvation temperature (°C)**	150	150	150
**Desolvation gas flow (L/h)**	550	550	550
**Cone gas flow (L/h)**	150	150	150
**Trap CE (V)**	7	5	32
**Transfer CE (V)**	7	7	7
**Trap gas flow**	6	4	4
**Trap DC bias (V)**	2	2	2
**Scan time (s)**	1	1	1
**Acquisition mass range (m/z)**	600-8000	50-8000	50-8000

Table S1. Main MS parameters used during the analysis of native proteins and ligand screening by TNT-MS.

identifier	structure	*m/z* ([M+H]$^+$)	target	name
a	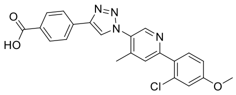	421.106	-	-
b	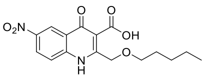	335.123	-	-
c	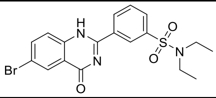	437.032	-	-
d	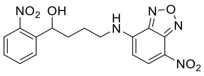	374.109	-	-
e	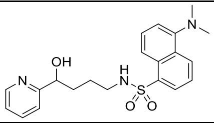	400.169	-	-
f	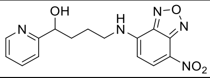	330.119	-	-
g	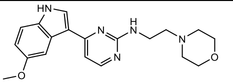	354.192	-	-
h	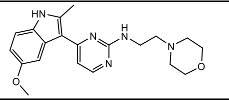	368.208	-	-
i	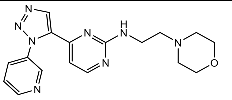	353.183	-	-
j	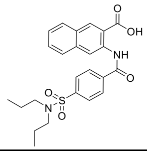	455.163	-	-
k	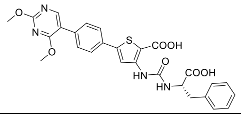	549.144	-	-
l	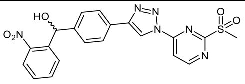	453.097	-	-
m	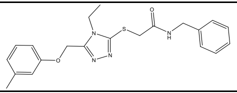	397.169	-	-
n	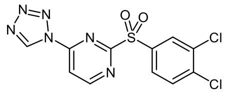	356.972	-	-
o	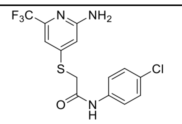	362.033	-	-
p	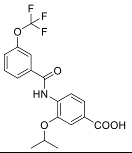	384.105	-	-
q	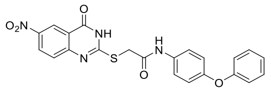	449.091	-	-

r	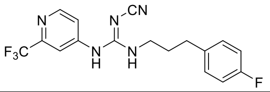	366.133	-	-
s	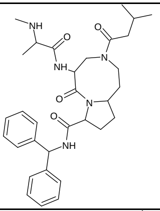	562.339	BIRC4	Xevinapant
t	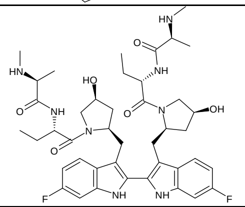	807.436	BIRC4	Birinapant
u	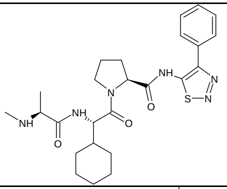	499.248	BIRC4	GDC-0152
v	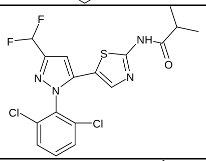	431.030	LIMK1	LIMKi3
w	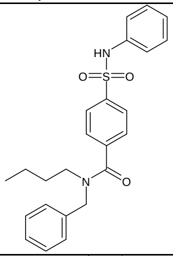	423.173	LIMK1	TH257
x	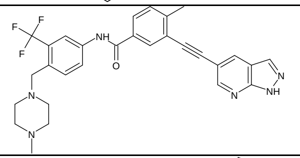	533.227	LIMK1 + AAK1 + STK17A	Olverembatinib
y	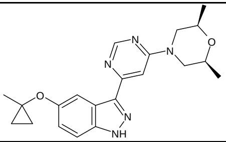	380.208	-	-
z	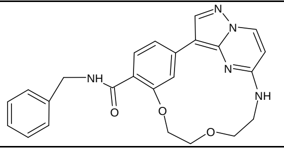	430.187	AAK1 + STK17A	CKJB68
IMID	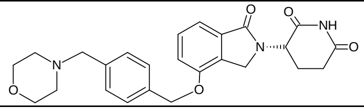	449.195	CRBN	CC-220

Table S2. Chemical structure and exact mass of protein ligands and negative controls used during the ligand screening experiment.

References

[1] L. Konermann, Addressing a Common Misconception: Ammonium Acetate as Neutral pH “Buffer” for Native Electrospray Mass Spectrometry, J. Am. Soc. Mass Spectrom. 28 (2017) 1827–1835. https://doi.org/10.1007/s13361-017-1739-3.

[2] K.J. Lee, J.S. Jordan, E.R. Williams, Is Native Mass Spectrometry in Ammonium Acetate Really Native? Protein Stability Differences in Biochemically Relevant Salt Solutions, Anal. Chem. 96 (2024) 17586–17593. https://doi.org/10.1021/acs.analchem.4c03415.

[3] G.J. Guimaraes, M.G. Bartlett, Managing nonspecific adsorption to liquid chromatography hardware: A review, Analytica Chimica Acta 1250 (2023) 340994. https://doi.org/10.1016/j.aca.2023.340994.

[4] S. Verma, N. Sharma, S. Kango, S. Sharma, Developments of PEEK (Polyetheretherketone) as a biomedical material: A focused review, European Polymer Journal 147 (2021) 110295. https://doi.org/10.1016/j.eurpolymj.2021.110295.

[5] M.P. Schwalm, L.M. Berger, M.N. Meuter, J.D. Vasta, C.R. Corona, S. Röhm, B.-T. Berger, F. Farges, S.M. Beinert, F. Preuss, V. Morasch, V.V. Rogov, S. Mathea, K. Saxena, M.B. Robers, S. Müller, S. Knapp, A Toolbox for the Generation of Chemical Probes for Baculovirus IAP Repeat Containing Proteins, Front. Cell Dev. Biol. 10 (2022) 886537. https://doi.org/10.3389/fcell.2022.886537.

[6] C.G. Kurz, F. Preuss, A. Tjaden, M. Cusack, J.A. Amrhein, D. Chatterjee, S. Mathea, L.M. Berger, B.-T. Berger, A. Krämer, M. Weller, T. Weiss, S. Müller, S. Knapp, T. Hanke, Illuminating the Dark: Highly Selective Inhibition of Serine/Threonine Kinase 17A with Pyrazolo[1,5- *a*]pyrimidine-Based Macrocycles, J. Med. Chem. 65 (2022) 7799–7817. https://doi.org/10.1021/acs.jmedchem.2c00173.

[7] T. Hanke, S. Mathea, J. Woortman, E. Salah, B.-T. Berger, A. Tumber, R. Kashima, A. Hata, B. Kuster, S. Müller, S. Knapp, Development and Characterization of Type I, Type II, and Type III LIM-Kinase Chemical Probes, J. Med. Chem. 65 (2022) 13264–13287. https://doi.org/10.1021/acs.jmedchem.2c01106.

[8] R.C.E. Deutscher, C. Meyners, M.L. Repity, W.O. Sugiarto, J.M. Kolos, E. Maciel, T. Heymann, T.M. Geiger, S. Knapp, F. Lermyte, F. Hausch, Discovery of fully synthetic FKBP12-mTOR molecular glues, Chem. Sci. 16 (2025) 4256-4263. https://doi.org/10.1039/D4SC06917J.
